# Supplementary material for: The portrayal of antimicrobial resistance in Bangladeshi newspapers during 2010–2021: Toward understanding the narrative
Source: PLoS One. 2024 May 31;19(5):e0304582. doi: 10.1371/journal.pone.0304582 (PMC11142656; doi:10.1371/journal.pone.0304582)
Supplement: S1 Appendix — (DOCX) [file pone.0304582.s001.docx]

**Annex**

**Annex Table 1: English and Bangla Dailies with Number of Circulations**

| **English Newspapers** | **Daily Circulation** | **Bangla Newspapers** | **Daily Circulation** |
| --- | --- | --- | --- |
| The Daily Star | 44,814 | Bangladesh Pratidin | 5,53,300 |
| The Financial Express | 39,010 | Prothom Alo | 5,01,800 |
| Daily Sun | 38,800 | Kaler Kantho | 2,90,200 |
| The Asian Age | 38,800 | Jugantor | 2,90,200 |
| The Daily Observer | 38,750 | The Daily Ittefaq | 2,90,200 |
| Dhaka Tribune | 38,700 | Janakantha | 2,75,000 |

(Source: Department of Films and Publications, Government of Bangladesh-2018)

**Annex Table 2: Electronic Search Words Used for Data Extraction**

| **Electronic Search Words** |
| --- |
| Antimicrobial resistance  Antibiotic resistance  Superbugs  Drug Resistance  Rational use of antibiotics  Infectious disease  AMR awareness  AMR stewardship  Microbial Resistance |
