# Supplementary material for: The portrayal of antimicrobial resistance in Bangladeshi newspapers during 2010–2021: Toward understanding the narrative
Source: PLoS One. 2024 May 31;19(5):e0304582. doi: 10.1371/journal.pone.0304582 (PMC11142656; doi:10.1371/journal.pone.0304582)
Supplement: Attachment — Submitted filename: Comments to author.docx [file pone.0304582.s004.docx]

Th authors have examined news stories on antimicrobial resistance in Bangladeshi newspapers published between to understand the narrative on the issues from the context of a developing country like Bangladesh. This is an interesting, time and relevant topic to investigated. It has high potential to contribute to the fields of mass media role in public health communication.

Title: The second part of the title of the article confusing. I recommend to write the title as follows: “The Portrayal of Antimicrobial Resistance in Bangladeshi Newspapers During 2010-2021: Toward understanding the narrative”

Abstract: The abstract does communicate the results of the study properly. I recommend to give deeper reflection of the results in the abstract

Introduction: The background should incorporate all aspect to the topic being investigated. But this paper lacks literature on media, its role in society in forming public opinion, raising awareness, it role in public health communication.

Methods: The method is not theoretically grounded. I recommend to use “Media Framing Theory” and “Agenda Setting Theory” to in extracting data, coding and analyzing the data.

Results: I found the results are communicated properly. But its improvement using more deeper and wider lenses of mass media theories and methods

Discussion: In this part, the authors should add more mass media related literatures to build the uniqueness of their findings

Conclusion: It seems good. But needs improvement
