# Supplementary material for: The portrayal of antimicrobial resistance in Bangladeshi newspapers during 2010–2021: Toward understanding the narrative
Source: PLoS One. 2024 May 31;19(5):e0304582. doi: 10.1371/journal.pone.0304582 (PMC11142656; doi:10.1371/journal.pone.0304582)
Supplement: Attachment — Submitted filename: Rebuttal Letter.docx [file pone.0304582.s005.docx]

| **Reviewer No.** | **Comment** | **Reply to Comment** |
| --- | --- | --- |
| **Data Availability** | | |
| 1 | Have the authors made all data underlying the findings in their manuscript fully available? | The database of the news articles is provided with their respective links. |
| **Title** | | |
| 1 | The second part of the title of the article is confusing. I recommend writing the title as follows: “The Portrayal of Antimicrobial Resistance in Bangladeshi Newspapers During 2010-2021: Toward understanding the narrative” | Acknowledged |
| **Abstract** | | |
| 1 | The abstract does communicate the results of the study properly. I recommend giving deeper reflection of the results in the abstract. | Necessary changes have been addressed. We have applied active voice and maintained throughout the document. We avoided phrases which did not provide specific information like “most of”; instead, we used specific numbers. |
| 2 | ‘disbursed’ seems intended to be ‘dispersed’ | Used “suggested” instead. |
| **Background** | | |
| 1 | The background should incorporate all aspects of the topic being investigated. But this paper lacks literature on media, its role in society in forming public opinion, raising awareness, its role in public health communication. | Acknowledged and addressed. We also made additional minor changes to improve the clarity of the manuscript that did not alter the content. |
| 2 | The point about ‘big pharma’ and media power (page 5; line 85) is a little weak, and the referenced material is only generally applicable. I suggest the authors integrate the point with local Bangladeshi context. For example, is direct-to-consumer drug advertising legal? And therefore, does it pose a smaller/larger issue than other nations? It is implied (page 20) that OTC is legal, so state this sooner. | [The Drugs (Control) Ordinance of 1982](http://bdlaws.minlaw.gov.bd/act-623/section-11969.html#:~:text=(%20Ordinance%20NO.,VIII%20OF%201982%20)&text=respect%20of%20drugs-,14.,Explanation.) in Bangladesh prohibits the publication of advertisements about drugs without the licensing authority's prior approval.   An Australian study monitored how the Big Pharma influences the doctors to prescribe their medicines. This information has been added. |
| **Method** | | |
| 1 | The method is not theoretically grounded. I recommend using “Media Framing Theory” and “Agenda Setting Theory” to in extracting data, coding and analyzing the data. | Acknowledged. We discussed both the theories as they both interacted in a step-wise model. |
| 2 | A comment may be needed in the data collection section, about the use (or not) of modifiers in search. For example, Boolean search (OR, AND, *) etc. Even a comment on whether these were possible on the newspaper websites or not would be valuable. If they were possible, terms like ‘superbugs’ in plural may have been better served by superbug*. These practices are common details of media research methods and might be important to note. | During this search, we did not use Boolean modifiers such as OR, AND, etc. The keywords were enough. That is because we wanted to capture all the articles that mentioned the keywords even for once. |
| **Results** | | |
| 1 | I found the results are communicated properly. But its improvement using more deeper and wider lenses of mass media theories and methods | We have discussed the issues in the Discussion section. We only tried to deliver the quantitative and qualitative findings. |
| **Discussions** | | |
| 1 | In this part, the authors should add more mass media related literatures to build the uniqueness of their findings | Acknowledged |
| 2 | ‘One Health’ is mentioned in relation to a speech (page 16) big is not discussed in the paper as an overall strategy. One health is becoming a popular framework for many: both in terms of writing about pharmacy operations (in humans and in industries like livestock), and also writing about media reporting and how AMR is framed. It may be useful to comment in this brief inclusion of One Health and how it differs from other regions. | Acknowledged |
| 2 | As well as the paper commenting on how Bangladesh might form a national strategy, the authors might like to also comment on what other nations can learn from the media reporting in Bangladesh. The matter of ‘over prescription’ is a universal topic in AMR media, but other areas seem more unique… It seems very significant that there is reporting on the ecosystem level of the problem! In other studies of AMR media, these complex issues are often overlooked in media. As the authors note, ‘scientific discovery’ based reporting is very common. The authors could note this difference and the significance. | Acknowledged |
| 2 | As the authors also note, ‘event’ based reporting is common. Additional connections and differences could be made here. There is research on media (including social media) and various ‘antibiotic awareness’ initiatives. Often these end up only reaching medical professionals and not impacting the community. So, it seems significant that Bangladeshi leaders are addressing AMR awareness in the media, and this is a point of difference from what is seen in international research. | We may not say that these initiatives are reaching the community. These events are for professionals, and the dailies only make an event-report. It will require more data to say that the leaders are directly addressing the public through the media on AMR. Therefore, the impact is as equal as in other countries. |
| 2 | While the authors do not need to explore linguistics (that is not the approach of the paper), it is notable that superbugs are not discussed much in the paper. Existing research suggests that superbugs are reported on in very dramatic ways and focus on individual suffering. The appearance (or not) of they may be worth commenting on. The main point is where a victim – for example, of MRSA infection – is situated. They are not consumers, but they suffer from the ramifications of AMR. Is this a feature of Bangladeshi media reporting? Do the authors perhaps see this type of story as part of the ‘provider end’ (the result of irrational prescription is the development of drug-resistance). Connections can be made here to Bouchoucha et al. (2019) Davis et al. (2020) and Capurro (2020) – all already cited in the paper. | Acknowledged |
| **Others (Minor Line notes)** | | |
| 2 | Line 87: ‘in a while’ – this needs to be clarified. Has SEA been evaluated to be currently at high risk, or is it consistently high risk? | Substituted with recent times. Therefore, currently. |
| 2 | Line 112-3: what is the ‘alignment’ with the WHO strategy? Is this just the date connection of 2010, or are the keywords shaped by the strategy? | Both, the date, and the keywords. |
| 2 | Line 126-36: an academic methodological source may be preferable to an online BBC article, to demonstrate a systematic approach to the differences between article types. | We added the reference from California State University San Marcos with BBC. As BBC is one of the pioneers of journalism worldwide, where many academicians and practicing journalists have contributions, we believe their addition to this issue holds equal value of an academic methodological perspective. |
| 2 | Line 139: ‘year wise numbers’ is a little unclear | Changed to “numbers of articles published in the newspapers per year” |
| 2 | Line 193: ‘sells’ should be ‘sales’ | Acknowledged |
| 2 | Line 251: language could be refined around the explanation of the study in India. 'Done in' is a little informal. | Changed to “conducted in” |
