# Supplementary material for: The portrayal of antimicrobial resistance in Bangladeshi newspapers during 2010–2021: Toward understanding the narrative
Source: PLoS One. 2024 May 31;19(5):e0304582. doi: 10.1371/journal.pone.0304582 (PMC11142656; doi:10.1371/journal.pone.0304582)
Supplement: Attachment — Submitted filename: Response to Reviewers.docx [file pone.0304582.s006.docx]

| **Reviewer No.** | **Comment** | **Reply to Comment** |
| --- | --- | --- |
| **Data Availability** | | |
| 1 | Have the authors made all data underlying the findings in their manuscript fully available? | The database of the news articles is provided with their respective links. |
| **Method** | | |
| 2 | The changes made to the Method section following review, are lacking detail. I note that the additional sentences (lines 136-140) explain the theories with nothing more than the name of the theory: ‘agenda setting theory will let us understand what news agenda has been set’ / ‘news framing theory will let us understand how news articles are framed’. This is too simple and does not show much engagement with either the theory or methods related to the theories. I believe the authors also need to think about the connection between the social psychology elements of such approaches and the media/content base of the paper, since these are quite different!  This is all quite necessary for two reasons: 1) If comments from the initial review about these theories is really being actioned, there should be deeper reflection here that is met by changes in the paper. 2) A broad journal like PLOS One has a very diverse readership, and the theories need to be explained in more detail.  I am also not sure that the single sources cited are the best choices for a clear picture of what exactly the theories do in the paper. The agenda setting article cited repeatedly is a conversation piece that relies on many other works by McCombs. The article refers to a book called ‘Setting the Agenda’ – perhaps this is a better source to reference? I think the authors need to reflect more on if/how these theories can actually add something to the paper, rather than just responding to the reviewer suggestions. | Agreed. Thanks. We feel that this feedback can really change the outlook of the people and as you said, the readers can easily connect with the research.  We added details on these two theories, and how these theories contributed to our understanding of the research question.   We added a sub-section named “Conceptual Framework” at the beginning of the “Methods” Section.  We cited two more references which discussed the intricacies of these two theories deeply. |
|  | I also maintain that the authors need to refining reference choices (at line 163-4). I agree with the authors comments that resources like the BBC are quality journalism. But the sources cited (including the CSUSM Library guide now added) are student-facing resources for media type identification; they do not then flow on detail use of the types for analytical research purposes. Other academic publications that discuss this (or similar) classification methods (and their relative importance) for media articles are necessary. I think this is very important, because new insights in the discussion about feature articles, jargon etc. (lines 362-371) give weight to distinctions between article types. Since this makes valuable about how information about AMR communication differs across these article types, the support of existing academic methods is needed. | Agreed.   We added academic references. Also, we added another reference of a similar study where the news articles were classified in a similar way. |
| **Discussion** | | |
| 2 | Since the new theories are being mentioned in the paper, they need to be more robustly reflected in the findings. ‘Agenda setting’ appears minimally (page 21) in reference to COVID. Which is somewhat prosaic and rushed. | Agreed. Added a section in Page 25. |
|  | ‘Infodemic’ is not defined (again remember PLOS readership is diverse) and I am unsure if has been entirely prevented by mass media… Regardless, how does this connect back to AMR, which is already not often reported on? | Infodemic definition included as “overwhelming quantity of information including incorrect and misleading information”. |
|  | Points about the status of readership (wealth) are also light on detail. What evidence is there – beyond intuition – that the economic class of a newspaper’s journalists and audiences equates to their education level? This work analysed the content of AMR reporting, not the readership demographics/understandings. These points then, seem more like future directions. | In Bangladesh, the English dailies are circulated in the urban areas, and historically, the readers are wealthier because of their exposure to English-medium schools. We did not think it would raise a question as it is a status quo for our country. However, we cited an article published in 2016 to resonate this phenomenon. |
|  | Points about personal stories seem important to consider, but the evidence here – ‘more reads’ (line 369) – is not referred to elsewhere in the paper. Did the authors have access to the publication metrics? If so, this needs to be described as part of the method. The authors also miss the opportunity to connect back to literature here. In sources cited already, the subject of human stories is mentioned. Linking back to this would aid the arguments made. | Some of the reports were shared on social media. We accessed whatever we could. We saw that there was more engagement in the Feature reports.   Agreed. Though it was not a part of the method, rather a side-exploration. However, for further studies regarding online engagement of news circulation, or even digital ethnography, we will recommend this feature.  Using both the theories discussed in the paper, we can understand how people can engage with news contents, and how we should formulate public health messages. |
| **Others (Minor Line notes)** | | |
|  | **Line 106:** why is the US based CDC being referred to as an international health body? | Though CDC is based in the US, it has operations worldwide. For example, in Bangladesh, they have a direct connection with the government both at strategy and operational level. Likewise, they have operations in many other countries as well in the areas of vaccination, vector-borne diseases, and infectious diseases. That is why we referred to the CDC as an international health body. |
|  | **Line 117:** sentence on WHO’s evaluation of AMR risk in South-East Asia remains confusing. Source date should be mentioned in text. Source it 10 years old, so cannot be seen as ‘recent’. What, definitionally, is a crisis? | The year is mentioned. As we wrote the article in early 2022, we did not have access to the 5^th^ GLASS report, and the Lancet report. That is why this surveillance data was the recent on Southeast Asia. We have excluded “recent” and mentioned the year. |
|  | **Line 125-7:** since the paper does not share research questions or hypothesis motivating the study, I would caution against the use of this kind of rhetorical question | Agreed. Sentence changed into assertive and now indicates to observatory action. |
|  | **Line 148-9:** point about ‘alignment’ with the WHO SEARO strategy remain unclear. Please clarify, for example, that the keyworks are a feature of the strategy. | The strategy includes mass media as a stakeholder for AMR awareness. That is why we mentioned about the alignment. |
|  | **Table 2:** following the changes elsewhere, ‘sells’ should be ‘sales’ | Agreed |
|  | **Line 330-4:** very long sentence with confusing conjunctions. Requires revising. | Changed into - The least reported recommendation for “provide end” by the news articles was availability of rapid diagnostic tests to differentiate between viral and bacterial causes of illness in health facilities at different levels. This can contribute significantly to reducing inappropriate use of antibiotics and be a cost-friendly and impactful initiative to help ensuring rational antibiotic prescribing |
|  | **Line 340-1:** there is quoted text from media here, please add reference. | We excluded the quote. It can be found in the dataset. However, we described the context. |
